# Draft Genome Sequence of *Anoxybacillus flavithermus* Strain 25, Isolated from the Garga Hot Spring in the Barguzin Valley, Baikal Region, Russian Federation

**DOI:** 10.1128/genomeA.01258-14

**Published:** 2014-12-04

**Authors:** Aleksey S. Rozanov, Alla V. Bryanskaya, Anastasia V. Kotenko, Tatiana K. Malup, Sergey E. Peltek

**Affiliations:** Institute of Cytology and Genetics, Siberian Branch of the Russian Academy of Sciences, Novosibirsk, Russian Federation

## Abstract

*Anoxybacillus flavithermus* strain 25 was isolated from a sediment sample from the Garga hot spring in the Barguzin Valley, Baikal Region, Russian Federation (54°19′3.72″N, 110°59′38.4″E). The sequenced and annotated genome is 2,838,680 bp and encodes 3,009 genes.

## GENOME ANNOUNCEMENT

The genus *Anoxybacillus* was described in 2000 by Pikuta and coworkers (1). This genus consisted of two species, one newly isolated *Anoxybacillus pushchinensis* and one existing *Bacillus flavothermus* (2), which was reclassified as *Anoxybacillus flavothermus*. To date, 19 species and two subspecies of *Anoxybacillus* have been reported (3). The members of this genus are not strict anaerobes, and most of the species have optimal growth in the temperature range of 50 to 65°C, are alkaliphilic or alkali tolerant, and are able to grow at a neutral pH (3).

Strain 25 was isolated from a sediment sample from the Garga hot spring (65°C) in the Barguzin Valley, Baikal Region, Russian Federation (54°19′3.72″N, 110°59′38.4″E).

*Anoxybacillus* strain 25 culture was cultivated in liquid medium containing 1% trypton, 0.5% yeast extract, and 3.5 M NaCl. Eight ml of cell culture were pelleted by centrifugation and resuspended in 75 µL of H_2_O by intense pipetting. DNA was isolated using the DNA purification kit (Fermentas). The Ion PGM Template OT2 400 and Ion PGM Template OT2 400 kits were used to create libraries for genome sequencing. Genome sequencing was performed on an IonTorrent platform (Applied Biosystems) using the Ion PGM Sequencing 400 kit in the SBRAS Sequencing Center.

*De novo* assembly of short reads into contigs was performed using MIRA version 4. Contigs shorter than 1,000 bp were deleted. A total of 212 contigs yielded a genome sequence 2,838,680 bp long, and the G+C content was 42.3%. Open-reading-frame prediction and automatic annotation were performed using NCBI PGAAP (http://www.ncbi.nlm.nih.gov/genome/annotation_prok). The complete genome sequence contained 3,009 genes, 23 rRNAs (5S, 16S, 23S), 61 tRNAs, and 1 ncRNA.

Phylogenetic analysis was performed using 16S rRNA sequences with the UPGMA algorithm implemented in MEGA version 6, and 16S rRNA sequences of *Anoxybacillus*-type strains were found using the StrainInfo (http://www.straininfo.net) and GenBank (http://www.ncbi.nlm.nih.gov/nucleotide) databases. According to phylogenetic analysis, *Anoxybacillus* strain 25 is most closely related to *Anoxybacillus flavithermus*.

### Nucleotide sequence accession numbers.

The draft genome sequence for *A. flavithermus* 25 has been deposited in DDBJ/EMBL/Genbank under the accession no. JPZO01000000. The 212 contigs have been deposited under accession numbers JPZO01000001 to JPZO01000212.
